# Comparing Tau PET Visual Interpretation with Tau PET Quantification, Cerebrospinal Fluid Biomarkers, and Longitudinal Clinical Assessment

**DOI:** 10.3233/JAD-230032

**Published:** 2023-05-16

**Authors:** Charles D. Chen, Maria Rosana Ponisio, Jordan A. Lang, Shaney Flores, Suzanne E. Schindler, Anne M. Fagan, John C. Morris, Tammie L.S. Benzinger

**Affiliations:** a Mallinckrodt Institute of Radiology, Washington University in St. Louis, St. Louis, MO, USA; b Department of Neurology, Washington University in St. Louis, St. Louis, MO, USA

**Keywords:** Alzheimer’s disease, cerebrospinal fluid, positron emission tomography, tauopathies

## Abstract

**Background:**

^18^F-flortaucipir PET received FDA approval to visualize aggregated neurofibrillary tangles (NFTs) in brains of adult patients with cognitive impairment being evaluated for Alzheimer’s disease (AD). However, manufacturer’s guidelines for visual interpretation of ^18^F-flortaucipir PET differ from how ^18^F-flortaucipir PET has been measured in research settings using standardized uptake value ratios (SUVRs). How visual interpretation relates to ^18^F-flortaucipir PET SUVR, cerebrospinal fluid (CSF) biomarkers, or longitudinal clinical assessment is not well understood.

**Objective:**

We compare various diagnostic methods in participants enrolled in longitudinal observational studies of aging and memory (*n* = 189, 23 were cognitively impaired).

**Methods:**

Participants had tau PET, Aβ PET, MRI, and clinical and cognitive evaluation within 18 months (*n* = 189); the majority (*n* = 144) also underwent lumbar puncture. Two radiologists followed manufacturer’s guidelines for ^18^F-flortaucipir PET visual interpretation.

**Results:**

Visual interpretation had high agreement with SUVR (98.4%)and moderate agreement with CSF p-tau181 (86.1%). Two participants demonstrated ^18^F-flortaucipir uptake from meningiomas. Visual interpretation could not predict follow-up clinical assessment in 9.52% of cases.

**Conclusion:**

Visual interpretation was highly consistent with SUVR (discordant participants had hemorrhagic infarcts or occipital-predominant AD NFT deposition) and moderately consistent with CSF p-tau181 (discordant participants had AD pathophysiology not detectable on tau PET). However, close association between AD NFT deposition and clinical onset in group-level studies does not necessarily hold at the individual level, with discrepancies arising from atypical AD, vascular dementia, or frontotemporal dementia. A better understanding of relationships across imaging, CSF biomarkers, and clinical assessment is needed to provide appropriate diagnoses for these individuals.

## INTRODUCTION

The pathological hallmarks of Alzheimer’s disease (AD) are amyloid-β (Aβ) plaques and misfolded hyperphosphorylated tau neurofibrillary tangles (NFTs) [1, 2]. *In vivo* evaluation of aggregated tau or associated pathophysiology in AD was first performed using immunoassays for cerebrospinal fluid (CSF) tau phosphorylated at position 181 (p-tau) [3]. Later, tau PET radiotracers were developed [4–6], along with methods for tau PET standardized uptake value ratio (SUVR) analyses [7, 8]. The first generation of tau PET radiotracers includes the arylquinoline derivatives ^18^F-THK5317 and ^18^F-THK5351, the pyrido-indole derivative ^18^F-flortaucipir, and the phenyl/pyridinyl-butadienyl-benzothiazole/benzothiazolium derivative ^11^C-PBB3. Among these, ^18^F-flortaucipir (Tauvid^TM^, Avid Radiopharmaceuticals) became the first to be approved by the United States Food and Drug Administration to estimate the density and distribution of aggregated tau NFTs in adult patients with cognitive impairment being evaluated for AD. Following the manufacturer’s guidelines for performing a visual interpretation of ^18^F-flortaucipir PET imaging involves identifying the presence or absence of contiguous radiotracer uptake greater than 1.65 times the cerebellar uptake in either the posterolateral temporal, occipital, or parietal/precuneus regions. This method differs greatly from most research procedures for automated quantification of tau PET imaging data, such as taking the volume-weighted mean standardized uptake value ratio (SUVR) in a temporal meta-region of interest (ROI) and comparing that to a cohort-defined threshold [7, 8]. These methodological differences may lead to disagreements between visual interpretation and SUVR quantification. In particular, the temporal meta-ROI often used in SUVR quantification does not contain any of the occipital or parietal/precuneus structures used in visual interpretation, and includes several medial temporal lobe structures ignored in visual interpretation. Additionally, in the clinic ^18^F-flortaucipir PET imaging is only indicated for use in adult patients with cognitive impairment who are being evaluated for AD, whereas in a research setting ^18^F-flortaucipir PET imaging is performed regardless of cognitive status, calling into question whether ^18^F-flortaucipir PET imaging is a reliable measure of NFT deposition during preclinical stages of AD. Tau pathophysiology can also be evaluated by measuring phosphorylated tau concentrations in the CSF, and several studies have provided additional evidence that tau PET is more strongly coupled to cognitive decline, whereas CSF p-tau181 is more tightly linked to preclinical AD [9–12]. Understanding where these three methods—tau PET visual interpretation, tau PET SUVR quantification, and CSF p-tau181 concentration—agree and differ may improve how we define AD NFT deposition and AD clinical diagnoses.

## MATERIALS AND METHODS

### Study participants

Participants selected for this study were enrolled in longitudinal observational studies of aging and memory at the Charles F. and Joanne Knight Alzheimer Disease Research Center (Knight ADRC, *n* = 189, of whom 23 were cognitively impaired, Table 1). All participants met the inclusion criteria of having a tau PET usable for visual reads, and an Aβ PET, MRI, and clinical and cognitive evaluation, all within 18 months; the majority of participants (*n* = 144) also underwent lumbar puncture within 18 months of their tau PET scan. The study was approved by the Washington University in St. Louis Human Research Protection Office and Institutional Review Board, and all participants or their designees signed an informed consent form.

**Table 1:** Participant characteristics

		Cognitively	Cognitively	Total
		normal	impaired
Number		166	23	189
Mean age in years (SD)		68.9±8.34	75.7±7.36	69.8±8.51
Female (%)		93 (56.0)	12 (52.2)	105 (55.6)
Race	White	147	23	170
	Black or African American	18	0	18
	Asian	1	0	1
Mean MMSE (SD)		29.2 (1.12)	26.0 (3.66)	28.8 (1.94)
CDR^®^	0	166	0	166
	0.5	0	16	16
	1	0	6	6
	2	0	1	1
Clinical diagnosis	Cognitively normal	166	0	166
	Uncertain dementia	0	9	9
	0.5 in memory only	0	1	1
	AD dementia	0	13	13
*APOE* genotype	2/2	1	0	1
	2/3	27	1	28
	2/4	6	1	7
	3/3	83	8	91
	3/4	42	11	53
	4/4	6	2	8
	Unknown	1	0	1
Tau PET temporal meta-ROI SUVR	Mean±SD	1.15±0.106	1.44±0.364	1.18±0.185
	[min, max]	[0.924, 1.882]	[1.024, 2.43]	[0.924, 2.43]
	Positive (%)	4 (2.41)	13 (56.5)	17 (8.99)
Tau PET visual interpretation	Positive (%)	6 (3.61)	14 (60.9)	20 (10.6)
Aβ PET (Centiloid)	Mean±SD	19.9±34.4	74.3±45.6	26.5±40.0
	Positive (%)	45 (27.1)	19 (82.6)	64 (33.9)

CDR^®^, Clinical Dementia Rating^®^; MMSE, Mini-Mental State Exam; SD, standard deviation.

### Clinical and cognitive assessment

Participants were assessed clinically and cognitively using the neuropsychological test battery from the Uniform Data Set (UDS) [13], which includes the Clinical Dementia Rating (CDR^®^) [14] and the Mini-Mental State Examination (MMSE) [15]. The CDR assesses three domains of cognition (memory, orientation, judgment, and problem solving) and three domains of function (community affairs, home and hobbies, personal care): scores from the six domains can either be summed to yield the CDR Sum of Boxes score, or passed to a lookup table to yield the CDR Global score.

### Tau PET acquisition

Participants were scanned on a Siemens Biograph 40 TruePoint (Siemens Healthineers). Participants received a single intravenous bolus injection (341±29.8 MBq) of ^18^F-flortaucipir (Tauvid^TM^, Avid Radiopharmaceuticals). Emission data were collected 80–100 min post injection. List-mode data were reconstructed using ordered subset expectation maximization with three iterations and 21 subsets. A low-dose CT scan preceded PET acquisition for attenuation correction.

### Tau PET SUVR

Reconstructed PET images were processed using the PET Unified Pipeline (https://github.com/ysu001/PUP) and coregistered to corresponding MR images [16, 17]. After segmenting MR images into ROIs using FreeSurfer version 5.3 [18], regional SUVRs were defined from the reconstructed PET images using a cerebellar gray reference region. The temporal meta-ROI SUVR was defined as the volume-weighted mean SUVR of the amygdala, entorhinal cortex, fusiform, parahippocampal, inferior temporal, and middle temporal ROIs [7, 8].

### Tau PET visual interpretation

Two radiologists with training in nuclear medicine (J.A.L. and M.R.P.) followed the manufacturer’s guidelines for ^18^F-flortaucipir PET visual interpretation of participant scans using MIM Encore (MIM Software). Reconstructed PET images were coregistered with corresponding MR images. A ROI was drawn around the whole cerebellum in the axial plane that maximizes its cross-sectional area. A color scale with a rapid transition at 1.65 times the mean cerebellar counts was defined. The temporal lobe was divided into the anterolateral, anterior mesial, posterolateral, and posterior mesial temporal quadrants by placing the horizontal crosshair posterior to the brainstem nuclei, and the vertical crosshair at the widest portion of the temporal pole. An image was considered positive if it showed contiguous activity above the rapid transition/cutoff in the cortical gray matter of the posterolateral temporal, occipital, or parietal/precuneus regions. An image was considered negative if it showed no activity above the cutoff in the cortical gray matter of the posterolateral temporal, occipital, or parietal/precuneus regions, or if it showed activity above the cutoff in the cortical gray matter restricted to the medial temporal, anterolateral temporal, and frontal regions. Off-target binding, which may be seen in the choroid plexus, striatum, and brainstem nuclei, and small foci of noncontiguous activity, which may be seen throughout the cortical gray matter, were not used when determining tau positivity. Radiologists were blinded to all other information about each participant. In addition to following the manufacturer’s guidelines for ^18^F-flortaucipir PET visual interpretation, in this study, radiologists also reported whether radiotracer activity was symmetric across left and right hemispheres and whether there was off-target binding in the choroid plexus, striatum, brainstem nuclei, or bone/meninges. Notable findings (such as incidental meningiomas) were also reported. Both radiologists determined off-target binding and incidental findings using MR imaging. Additionally, incidental findings were confirmed with a neuroradiologist (T.L.S.B.).

### Aβ PET

Participants were scanned on either a Siemens Biograph 40 TruePoint, Biograph mMR, or Biograph Vision 600 (Siemens Healthineers). Participants received either a single intravenous bolus injection (539±159 MBq) of ^11^C-Pittsburgh compound B (PiB) or (369±22.4 MBq) of ^18^F-florbetapir (Amyvid^TM^, Avid Radiopharmaceuticals). Emission data were either collected 30–60 min post injection (^11^C-PiB) or 50–70 min post injection (^18^F-florbetapir). Reconstructed PET images were formed and pre-processed in the same manner as tau PET. An Aβ PET SUVR was defined for each radiotracer [16, 17] and standardized to the Centiloid scale [19, 20].

### MR acquisition

Participants were scanned on either a Siemens Biograph mMR or Magnetom Vida (Siemens Healthineers). Across all scanners, T1-weighted head MR images were acquired using a magnetization prepared rapid gradient echo (MPRAGE) generalized autocalibrating partial parallel acquisition (GRAPPA) sequence using a repetition time = 2300 ms, echo time = 2.95 ms, flip angle = 9°, at 1.1×1.1×1.2 mm^3^ voxel resolution.

### CSF

CSF was collected under standardized operating procedures. Participants underwent lumbar puncture in the morning following overnight fasting and 20–30 ml of CSF was collected in a 50 ml polypropylene tube via gravity drip using an atraumatic Sprotte 22-gauge spinal needle. CSF samples were kept on ice and centrifuged at low speed within 2 h of collection, then transferred to another 50 ml tube to remove cells. CSF was aliquoted at 500μl into polypropylene tubes and stored at –80°C. Concentrations of CSF p-tau181, Aβ_42_, and Aβ_40_ were measured by chemiluminescent enzyme immunoassay using a fully automated platform (LUMIPULSE G1200, Fujirebio) according to the manufacturer’s specifications.

### Statistical analyses

Cutoffs for binarizing tau PET, Aβ PET, CSF p-tau181, and CSF Aβ_42_/Aβ_40_ values were determined by fitting a two-component univariate Manly mixture model [21] in R software [22] to all relevant baseline PET SUVR or CSF measurements available in the Knight ADRC Data Freeze 17 (Supplementary Table 1) and finding the decision boundary. Manly mixture modeling was used to account for possible severe skewness in the data that would be difficult to model using Gaussian mixture modeling, and to account for skewness that can vary from component to component, which would be impossible to model using log or Box-Cox transformations [21]. Cohen’s kappa (κ) was used to measure inter-rater reliability between the two radiologists’ tau PET visual interpretations, as well as between tau PET visual interpretation and tau PET SUVR quantification, and between tau PET visual interpretation and CSF p-tau181 concentration.

## RESULTS

### Study participants

Overall, participants were on average (±standard deviation) 69.8±8.51 years old, most were cognitively normal with a global Clinical Dementia Rating (CDR^®^) [14] of 0 (*n* = 166/189, 87.8%) and most did not carry the *APOE* ɛ4 allele (*n* = 120/188, 63.8%) (Table 1). Cognitively normal participants had a mean tau PET temporal meta-ROI SUVR of 1.15±0.106 and a mean Centiloid of 19.9±34.4. Cognitively impaired participants (*n* = 23/189, 12.2%) had a clinical diagnosis of either uncertain dementia (*n* = 9), a CDR = 0.5 in memory only (*n* = 1), or AD dementia (*n* = 13). They also had a mean tau PET temporal meta-ROI SUVR of 1.44±0.364 and a mean Centiloid of 74.3±45.6.

The following quantitative cutoffs were identified through statistical modeling and are used to determine biomarker positivity in the remainder of the analyses: tau PET temporal meta-ROI SUVR cutoff = 1.32, Aβ PET (Centiloid) cutoff = 21.6, CSF p-tau181 cutoff = 58.1 pg/ml, and CSF Aβ_42_/Aβ_40_ cutoff = 0.0737.

### Tau PET visual interpretation and tau PET SUVR

**Fig. 1:**
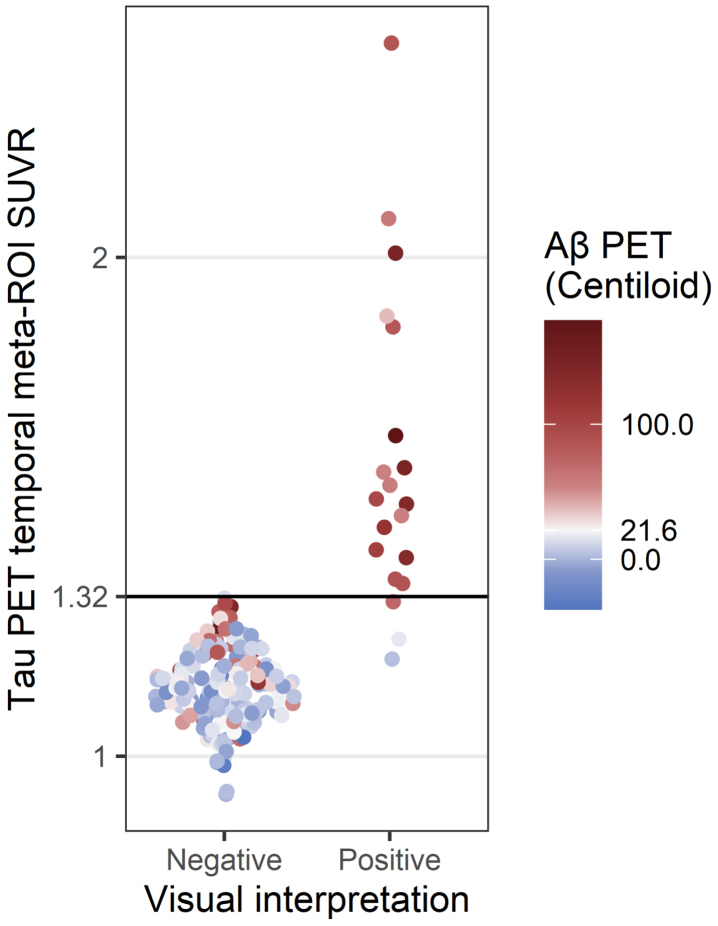
Comparison of tau PET visual interpretation with tau PET SUVR. Each PET study was assessed by visual interpretation using the manufacturer’s guidelines to determine positivity (x-axis) and by temporal meta-ROI SUVR analysis using a cutoff of SUVR = 1.32 to determine positivity (y-axis). The color indicates the Aβ PET status for each case (positive Aβ PET, red; negative Aβ PET, blue; cutoff = 21.6 Centiloids).

Of the 189 ^18^F-flortaucipir PET images, 20 (10.6%) were read as positive by both radiologists. Both radiologists also read 169 images as negative and thus agreed on the overall visual interpretation of each image in the current study (*n* = 189/189, 100%, κ= 1). Agreement between visual interpretation and SUVR quantification was high (*n* = 186/189, 98.4%, κ= 0.910) (Fig. 1).

**Fig. 2:**
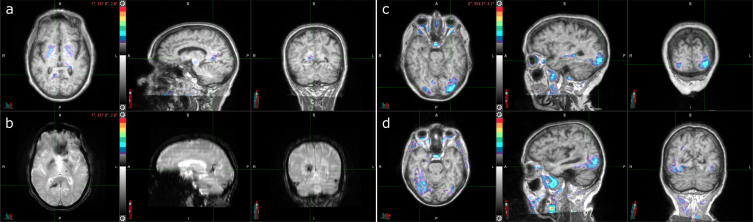
Three cases with tau-positive visual interpretations, but tau-negative SUVRs. a) Tau PET coregistered with MRI of a male participant in his 80s with elevated right precuneus uptake. b) Corresponding T2*-weighted MRI showing a hypointensity (indicated by the crosshair) that colocalizes with the elevated right precuneus uptake from (a). c) Tau PET coregistered with MRI of a female participant in her 70s with elevated occipital lobe uptake, left greater than right. d) Tau PET coregistered with MRI of a female participant in her 70s with elevated posterolateral temporal, occipital, and parietal/precuneus lobe uptake, right greater than left.

The three participants who had discordant results between visual interpretation and SUVR quantification all had tau-positive visual interpretations and tau-negative SUVRs. One participant (Fig. 2a) demonstrated elevated ^18^F-flortaucipir uptake in the right precuneus and was Aβ PET, CSF Aβ_42_/Aβ_40_, and CSF p-tau181 negative (Table 2). Additional MR imaging revealed a hypointensity on T2*-weighted MRI that colocalized with the elevated right precuneus radiotracer uptake on ^18^F-flortaucipir PET, suggesting a hemorrhagic infarct to be the cause of elevated radiotracer uptake instead of AD NFT deposition (Fig. 2b). Upon review of the additional T2*-weighted MR imaging, the readers also revised their interpretation of the image to be tau negative.

**Table 2:** AD biomarker status for cases with positive tau PET visual interpretation but negative tau PET SUVR analysis

	Age	Sex	*APOE*	CDR^®^	Aβ PET	Tau PET	CSF	CSF p-tau181
					(Centiloid)	(SUVR)	Aβ_42_/Aβ_40_	(pg/ml)
Parietal/precuneus hemorrhagic infarct	80s	Male	3/4	0	3.87	1.19	0.0975	21.6
Left occipital	70s	Female	3/4	0	17.0⟶50.0^*^	1.23	**0.0523**	**69.2**
Right occipital	70s	Female	3/3	0.5	**72.1**	1.31	**0.0493**	**63.5**

Numbers in bold denote positive biomarker status. ^*^This participant had a Centiloid = 17.0 (below cutoff) approximately one year before their tau PET visit, and a Centiloid = 50.0 (above cutoff) approximately two years after their tau PET visit.

The other two participants (Fig. 2c, d) demonstrated lateralized occipital uptake, with greater uptake in either the left (Fig. 2c) or right (Fig. 2d), and were Aβ PET, CSF Aβ_42_/Aβ_40_, and CSF p-tau181 positive (Table 2). The participant with greater left occipital uptake than right, likely has an occipital-predominant form of AD tau pathology [23].

The participant with greater right occipital uptake than left also had posterolateral temporal and parietal/precuneus uptake. The temporal meta-ROI SUVR was borderline negative, suggesting that, perhaps due to the lateralized uptake, the SUVR was artificially low for this case.

### Incidental findings

**Fig. 3:**
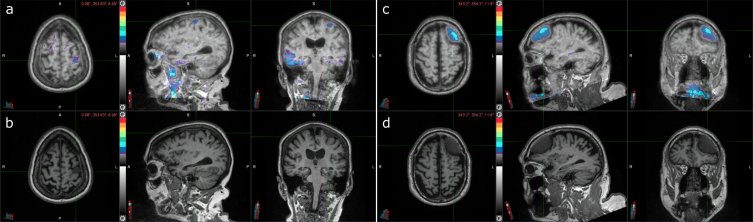
Two cases of incidental meningioma with tau PET uptake. a) Tau PET coregistered with MRI of a female participant in her 70s with a left frontal posterior meningioma (indicated by the crosshair) with tau radiotracer uptake. b) Corresponding MRI image. c) Tau PET coregistered with MRI of a male participant in his 70s with a left frontal meningioma (indicated by the crosshair) with tau radiotracer uptake. d) Corresponding MRI image.

In terms of incidental findings, frontal meningiomas were identified in two participants. One participant had a meningioma in their left posterior frontal lobe (Fig. 3a, b); the other participant had it in their left frontal lobe (Fig. 3c, d). Both meningiomas had elevated levels of radiotracer uptake. The first participant also had elevated right posterolateral temporal uptake and tau-positive visual interpretation and SUVR and was Aβ PET positive (Table 3). The other participant had tau-negative visual interpretation and SUVR and was Aβ PET negative.

**Table 3:** AD biomarker status for cases with incidental meningioma

	Age	Sex	*APOE*	CDR^®^	Aβ PET	Tau PET	CSF	CSF p-tau181
					(Centiloid)	(SUVR)	Aβ_42_/Aβ_40_	(pg/ml)
Left posterior frontal meningioma	70s	Female	2/4	0	**177**	**1.64**	0.0481^*^	49.9^*^
Left frontal meningoma	70s	Male	2/3	0	8.94	1.16	0.0848^*^	30.3^*^

Numbers in bold denote positive biomarker status. ^*^CSF lumbar punctures were performed approximately 10 years prior to tau PET.

### CSF p-tau181

**Fig. 4:**
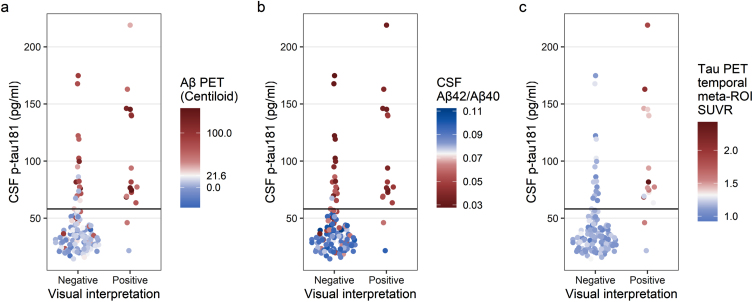
Comparison of tau PET visual interpretation with CSF p-tau181 concentration. Each participant is plotted by visual interpretation (x-axis) and CSF p-tau181 concentration (y-axis); participants with p-tau181 ≥58.1 pg/ml were considered positive. In (a), the color indicates the Aβ PET status for each participant (positive Aβ PET, red; negative Aβ PET, blue; cutoff = 21.6 Centiloid). In (b), the color indicates the CSF Aβ_42_/Aβ_40_ status for each participant (positive CSF Aβ_42_/Aβ_40_, red; negative CSF Aβ_42_/Aβ_40_, blue; cutoff = 0.0737). In (c), the color indicates the tau PET temporal meta-ROI SUVR status for each participant (positive tau PET, red; negative tau PET, blue; cutoff = 1.32 SUVR).

Agreement between visual interpretation and CSF p-tau181 was moderate (*n* = 124/144, 86.1%, κ= 0.526, Table 4). Two participants had tau-positive visual interpretations but were CSF p-tau181 negative (Fig. 4a, b). One participant was previously identified as having a tau-positive visual interpretation but tau-negative SUVR (the same case as in Fig. 2a, b). The other participant demonstrated posterolateral temporal uptake in both hemispheres and was Aβ PET and CSF Aβ_42_/Aβ_40_ positive. In addition, 18 participants had tau-negative visual interpretations but were CSF p-tau181 positive (Fig. 4a, b). These cases were mostly Aβ PET positive (*n* = 14/18, 77.8%) and/or CSF Aβ_42_/Aβ_40_ positive (*n* = 17/18, 94.4%).

**Table 4:** Participant characteristics for those who underwent lumbar puncture

		Cognitively	Cognitively	Total
		normal	impaired
Number		126	18	144
Mean age in years (SD)		68.6±8.32	76.1±7.84	69.5±8.60
Female (%)		70 (48.6)	10 (55.6)	80 (55.6)
Race	White	114	18	132
	Black or African American	11	0	11
	Asian	1	0	1
Mean MMSE (SD)		29.3 (1.07)	25.7 (3.90)	28.8 (2.06)
CDR^®^	0	126	0	126
	0.5	0	12	12
	1	0	5	5
	2	0	1	1
Clinical diagnosis	Cognitively normal	126	0	126
	Uncertain dementia	0	6	6
	AD dementia	0	12	12
*APOE* genotype	2/2	1	0	1
	2/3	22	1	23
	2/4	3	1	4
	3/3	62	7	69
	3/4	31	7	38
	4/4	6	2	8
	Unknown	1	0	1
Tau PET temporal meta-ROI SUVR	Mean±SD [min, max]	1.15±0.108	1.47±0.367	1.19±0.194
		[0.924, 1.882]	[1.042, 2.43]	[0.924, 2.43]
	Positive (%)	3 (2.38)	11 (61.1)	14 (9.72)
Tau PET visual interpretation	Positive (%)	5 (3.97)	12 (66.7)	17 (11.8)
Aβ PET (Centiloid)	Mean±SD	19.0±32.2	80.3±46.7	26.7±39.8
	Positive (%)	34 (27.0)	16 (88.9)	50 (34.7)
CSF p-tau181	Mean±SD	42.6±30.4	88.7±42.6	48.4±35.5
	Positive (%)	19 (15.1)	14 (77.8)	33 (22.9)
CSF Aβ_42_/Aβ_40_	Mean±SD	0.0777±0.0217	0.0502±0.0196	0.0743±0.0233
	Positive (%)	41 (32.5)	16 (88.9)	57 (39.6)

### Clinical and cognitive assessment

Six participants were assessed at baseline to be cognitively normal but tau-positive on visual interpretation (Table 5). One participant (Case 1) was previously mentioned to have PET radiotracer uptake colocalized to a parietal/precuneus hypointensity on T2*-weighted MRI and no other positive AD biomarkers (Fig. 2 and Table 2). The remaining five participants were all Aβ PET positive. No participant reliably converted from cognitively normal to AD dementia. One participant (Case 2) did convert to AD dementia at their three-year follow up but was reassessed to have a clinical diagnosis of uncertain dementia, more specifically, possible non-AD dementia of vascular origin at their five-year follow up. Another participant (Case 4) converted to AD dementia at their two-year follow up but was reassessed to have frontotemporal dementia (FTD) at their four-year follow up.

**Table 5:** Cognitively normal participant follow up

	Age	Sex	*APOE*	Baseline tau PET temporal meta-ROI	Baseline tau PET visual interpretation	Asymmetry	Baseline Aβ PET (Centiloid)	Yearly follow-up clinical diagnosis				
								1	2	3	4	5
Baseline cognitively normal and tau PET visual interpretation positive												
1^*^	80s	Male	3/4	1.19	**Positive**	Right	3.87	CN	CN	CN	CN	CN
2	70s	Male	2/3	**1.35**	**Positive**	Left	**86.6**		CN	AD	AD	UD
3^**^	70s	Female	3/4	1.23	**Positive**	Left	17.0⟶**50.0**	CN	CN	CN	CN	CN
4	80s	Male	3/3	**1.88**	**Positive**	Left	**37.6**	UD	AD	AD	FTD
5	80s	Male	3/4	**1.54**	**Positive**		**57.3**	CN			
6^***^	70s	Female	2/4	**1.64**	**Positive**	Right	**177**	CN	CN	CN	
Baseline cognitively normal (and tau PET visual interpretation negative), but converts at follow up												
7	70s	Female	3/4	1.22	Negative		–2.35	AD	CN	CN	
8	80s	Female	3/4	1.21	Negative		**120**	CN	AD	AD	
9	50s	Male	3/3	0.93	Negative		3.38			UD	AD
10^****^	70s	Male	2/3	1.16	Negative		8.94	AD	CN	CN	

Numbers in bold denote positive biomarker status. AD, Alzheimer’s disease (dementia); CN, cognitively normal; FTD, frontotemporal dementia; UD, uncertain dementia. ^*^Same case as the “Parietal/precuneus hemorrhagic infarct” case in Table 2. ^**^Same case as the “Left occipital” case in Table 2. ^***^Same case as the “Left posterior frontal meningioma” case in Table 3. ^****^Same case as the “Left frontal meningioma” case in Table 3.

Four participants were assessed at baseline to be cognitively normal and tau-negative on visual interpretation but would convert to AD dementia at follow up (Table 5). Two participants (Case 7 and Case 10) converted to AD dementia at their one-year follow ups but were reassessed as cognitively normal at their two-year follow ups. The remaining two participants (Case 8 and Case 9) converted to AD dementia at their second- and fourth-year follow ups, respectively, but only Case 2 demonstrated Aβ PET positivity at baseline.

Twenty-three participants were assessed at baseline to have cognitive impairment (Table 6). Nine of these participants received a clinical assessment of uncertain dementia and two of the nine had a baseline tau-positive visual assessment. Both cases (Case 2 and Case 4) converted to AD dementia by their first- and second-year follow ups, respectively and were both Aβ PET positive. Nonetheless, three cases with a tau-negative visual interpretation at baseline (Case 5, Case 7, and Case 8) converted to AD dementia at their two-, two-, and three-year follow ups, respectively, although Case 5 was reassessed to be cognitively normal at their five-year follow up.

**Table 6:** Cognitively impaired participant follow up

	Age	Sex	*APOE*	Baseline tau PET temporal meta-ROI	Baseline tau PET visual interpretation	Asymmetry	Baseline Aβ PET (Centiloid)	Yearly follow-up clinical diagnosis				
								1	2	3	4	5
Baseline uncertain dementia												
1	60s	Male	3/4	1.11	Negative		**51.0**		UD	UD	CN	UD
2	70s	Female	3/4	**1.86**	**Positive**		**82.7**	AD	AD	AD	
3	60s	Female	3/3	1.14	Negative	Right	**37.6**	CN		CN	CN	CN
4^*^	70s	Female	3/3	1.31	**Positive**	Right	**72.1**	UD	AD	AD	AD
5	70s	Male	3/4	1.10	Negative	Right	**77.9**	CN	UD	AD	UD	CN
6	70s	Female	2/3	1.16	Negative		–25.7	UD			
7	80s	Male	2/4	1.04	Negative		9.49	CN	AD		
8	70s	Female	4/4	1.29	Negative		**80.1**		UD	AD	AD
9	70s	Male	3/4	1.02	Negative	Right	19.2	CN	CN	CN	CN
Baseline 0.5 in memory only												
10	80s	Male	3/3	1.07	Negative		12.2	CN	CN		
Baseline AD dementia												
11	70s	Female	3/4	**1.57**	**Positive**		**54.7**	AD	AD		
12	60s	Male	3/3	**2.43**	**Positive**		**85.7**				
13	70s	Female	3/4	**2.08**	**Positive**		**60.1**	AD	AD	AD	AD
14	80s	Male	3/3	**1.46**	**Positive**		**121**	AD	AD		
15	80s	Male	3/4	**1.35**	**Positive**		**87.5**	AD	AD	AD	AD
16	70s	Female	4/4	**1.58**	**Positive**	Left	**145**	AD	AD		
17	70s	Female	3/4	**1.52**	**Positive**		**97.2**	AD			
18	70s	Female	3/4	1.18	Negative		**62.8**	FTD			
19	70s	Male	3/4	**1.40**	**Positive**	Right	**134**	AD	AD	AD	AD
20	80s	Male	3/4	**1.51**	**Positive**		**138**	AD	AD		
21	80s	Female	3/3	**1.41**	**Positive**	Right	**106**	CN	CN		
22	70s	Female	3/4	**1.48**	**Positive**	Left	**55.9**	AD	AD		
23	50s	Male	3/3	**2.01**	**Positive**		**145**				

Numbers in bold denote positive biomarker status. AD, Alzheimer disease (dementia); CN, cognitively normal; FTD, frontotemporal dementia; UD, uncertain dementia. *Same case as the “Right occipital” case in Table 2.

Thirteen of the 23 participants with baseline cognitive impairment received a clinical assessment of AD dementia. All 13 participants were Aβ PET positive (Table 6). Twelve of these participants had tau-positive visual interpretation; the remaining participant (Case 18) was tau PET negative, but at their one-year follow up had their clinical assessment changed to FTD. Additionally, Case 21 was tau PET positive, but was reassessed to be cognitively normal at their one- and two-year follow ups.

### Conclusions

^18^F-flortaucipir PET visual interpretation was found to be consistent between readers in this study (*n* = 189/189, 100%, κ= 1) and highly consistent with SUVR quantification (*n* = 186/189, 98.4%, κ= 0.910), suggesting these two approaches to determining tau PET positivity give similar results despite their different methodologies. However, three participants had discordant visual interpretations and SUVRs, likely due to a hemorrhagic infarct with elevated radiotracer uptake, an atypical, occipital-predominant presentation of AD NFT deposition, and a highly lateralized presentation of AD NFT deposition, respectively. These cases suggest the need for MR imaging to accompany ^18^F-flortaucipir PET visual interpretation, and the need to consider regions outside the temporal meta-ROI for SUVR quantification.

Among non-AD sources of ^18^F-flortaucipir uptake, the most studied is off-target binding in the choroid plexus, striatum, brainstem, and bone/meninges [24, 25]. In this study, off-target binding did not mimic the appearance of the AD tau pattern when assessed by visual readers, nor did it cause any tau PET temporal meta-ROI SUVR to be falsely positive when compared to visual interpretation. However, we observed two other sources of off-target binding that were not mentioned in the manufacturer’s guidelines for ^18^F-flortaucipir PET visual interpretation and which can potentially confound tau PET interpretations: hemorrhagic infarcts and meningiomas. The hemorrhagic infarct case was the case previously described as having a tau-positive visual interpretation and a tau-negative SUVR quantification. The two meningioma cases demonstrated elevated levels of radiotracer uptake in the frontal lobe, which is immaterial when assessing tau PET positivity by visual interpretation, but meningiomas in the posterolateral temporal, occipital, or parietal/precuneus regions might plausibly interfere with visual interpretation and SUVR quantification.

^18^F-flortaucipir PET visual interpretation was found to be moderately consistent with CSF p-tau181 (*n* = 124/144, 86.1%, κ= 0.526). Most discordant cases (*n* = 18/20) are amyloid-positive and CSF p-tau181 positive, but tau-negative on visual interpretation. This suggests that the discordance between ^18^F-flortaucipir PET and CSF p-tau181 may be attributed to participants with early changes in AD pathophysiology. Moloney and colleagues have found in an autopsy study that p-tau181, 205, 217, and 231 fluid biomarker sites are present in the early stages of NFT maturity [26]. Wennström and colleagues have found that p-tau217 can be found within NFTs and neuropil threads along with p-tau181, 231, 202, 202/205, and 369/404, and that p-tau217 area fraction correlated with antemortem plasma p-tau217 in individuals with confirmed Aβ plaque pathology [27]. Furthermore, plasma p-tau and CSF p-tau have been shown to be strongly correlated [28–30]. Taken together, these findings suggest a possibility that CSF p-tau181 is tracking changes in AD pathophysiology that occur earlier than the more advanced stages of NFT aggregation that ^18^F-flortaucipir PET may be more sensitive to.

When interpreting tau PET visual interpretation alongside clinical diagnosis after the study (both visual interpretation and clinical diagnosis were performed independently) a few relationships between the two kinds of AD diagnoses were remarkable. First, a baseline tau-positive visual interpretation in participants who were cognitively normal at baseline did not reliably predict conversion to AD dementia at follow up. If anything, tau PET positivity in cognitively normal participants was more likely to be either a sign of atypical AD, of related dementias (vascular dementia or FTD), or of resilience to AD dementia. Second, a baseline tau-negative visual interpretation in participants who were cognitively normal at baseline did not rule out conversion to AD dementia at follow up. Four cases were found to demonstrate conversion to AD dementia at follow up under these circumstances, although two of these were later reassessed to be cognitively normal. Third, baseline tau PET positivity in cognitively impaired participants did not guarantee a diagnosis of AD dementia at follow up: one participant was assessed to be cognitively normal at follow up even under these circumstances and another was reassessed to have FTD. Finally, baseline tau PET negativity in cognitively impaired participants cannot be used to rule out conversion to AD dementia at follow up: three such participants converted to AD dementia at their follow up visits, respectively, although one was reassessed to be cognitively normal at a later date.

A bias of the current study lies in the inclusion of cognitively normal participants. In a clinical setting, ^18^F-flortaucipir PET is indicated for use in patients with cognitive impairment. Two of the three cases discordant between visual interpretation and SUVR quantification in this study were from cognitively normal participants and would not warrant the use of ^18^F-flortaucipir visual interpretation in a clinical setting to begin with. Six of the 20 cases discordant between visual interpretation and CSF p-tau181 quantification were from cognitively normal participants and also would not warrant the use of ^18^F-flortaucipir visual interpretation in a clinical setting. Furthermore, the inclusion of cognitively normal participants, who represent the majority of the cases studied, also introduces a negative case bias, as they also represent a majority of the tau PET negative cases. Since most of these cases have tau PET SUVRs much lower than the SUVR positivity threshold, the agreement between visual interpretation and SUVRs is higher in the current study compared to a more challenging study comparing exclusively borderline cases. Nonetheless, exploring tau positivity in cognitively normal participants in this study identified individuals who have atypical AD tau and clinical progression.

The current study is focused on ^18^F-flortaucipir PET, and its conclusions do not necessarily apply to other tau PET radiotracers, which may have their own distinctive characteristics regarding off-target binding and sensitivity, which need to be accounted for on a tracer-by-tracer basis in studies of visual interpretation guidelines [31].

Future studies may also explore the discordances between tau PET visual interpretation and tau PET SUVR more thoroughly by investigating those participants with questionable or very mild dementia. Future studies may also explore the consequences of using regions of interest beyond the temporal meta-ROI used in the current study, such as the MUBADA [32]. These alternative ROIs may be appropriate for increasing concordance between visual interpretation and SUVR quantification, especially if crucial differences between the two lie in elevated radiotracer uptake outside the temporal meta-ROI. That said, the most direct way of harmonizing visual interpretation and SUVR quantification may be to construct an entirely new ROI for SUVR quantification that covers the critical regions in visual interpretation (posterolateral temporal, occipital, and parietal/precuneus regions) and develop a quantification method sensitive to contiguous uptake within this ROI.

In conclusion, ^18^F-flortaucipir PET visual interpretation can identify atypical AD NFT deposition that may be missed by SUVR quantification depending upon the regions of interest used. However, while the manufacturer’s guidelines for ^18^F-flortaucipir PET visual interpretation address non-AD sources of uptake such as off-target binding, they do not address other non-AD sources of uptake such as hemorrhagic infarcts and meningiomas. Temporal meta-ROI SUVR was highly concordant with visual interpretation for the cohort considered in this study. However, SUVR analyses could not detect lateralized occipital-predominant AD NFT deposition because the occipital lobe falls outside the temporal meta-ROI. CSF p-tau181 concentration was moderately concordant with visual interpretation and enabled detection of early changes in AD pathophysiology associated with tau hyperphosphorylation. However, these changes cannot be seen on PET. Finally, a positive visual interpretation did not make a follow up diagnosis of AD dementia inevitable, and a negative visual interpretation did not exclude the possibility of a follow up diagnosis of AD dementia. Additional work is needed to understand how multiple AD PET and CSF biomarkers might conceivably be used in tandem in a clinical setting alongside AD clinical evaluation in order to correctly diagnose and treat all individuals, not just those who demonstrate AD biomarker and clinical findings concordant with group-level trends.

## Supplementary Material

Supplementary Material

## Data Availability

The datasets generated during and/or analyzed during the current study are available from the corresponding author on reasonable request.
