# Supplementary material for: Comparing Tau PET Visual Interpretation with Tau PET Quantification, Cerebrospinal Fluid Biomarkers, and Longitudinal Clinical Assessment
Source: J Alzheimers Dis. 2023 May 16;93(2):765–77. doi: 10.3233/JAD-230032 (PMC10200228; doi:10.3233/JAD-230032)
Supplement: Supplementary Material [file jad-93-jad230032-s001.pdf]

Supplementary Material

Comparing Tau PET Visual Interpretation with Tau PET Quantification, Cerebrospinal Fluid Biomarkers, and Longitudinal Clinical Assessment

Supplementary Table 1. Participant characteristics for the Knight ADRC Data Freeze 17

		Tau PET	Aβ PET (^{11}C-PiB)	CSF
**Number**		494	738	1183
**Mean age in years (SD)**		70.4 (8.2)	68.6 (9.5)	69.2 (9.5)
**Female (%)**		274 (55.5)	425 (57.6)	640 (54.1)
**Mean MMSE (SD)***		28.7 (2.1)	28.7 (1.9)	28.1 (2.8)
**CDR®, n**	**0**	415	608	862
	**0.5**	60	98	241
	**1**	17	21	75
	**2**	1	0	5
	**Unknown***	1	11	0
***APOE* genotype, n**	**2/2**	1	3	4
	**2/3**	56	81	113
	**2/4**	11	19	32
	**3/3**	236	369	566
	**3/4**	154	210	379
	**4/4**	27	42	75
	**Unknown***	9	14	0

*Not all participants with AD biomarkers had clinical and cognitive evaluations within 18 months, or known *APOE* genotypes; however, this does not warrant their exclusion when defining AD biomarker positive cutoffs, which in our study only rely on the biomarker values themselves.
